# The 32nd Brazilian Society of Virology (SBV) 2021 Annual Meeting

**DOI:** 10.3390/v14030644

**Published:** 2022-03-20

**Authors:** Maite Freitas Silva Vaslin, Alessandra Alevato Leal, Larissa Mayumi Bueno, Cíntia Bittar, Gabriela Fabiano de Souza, Karine Lourenço, Gustavo Peixoto Duarte da Silva, Maria Isabel Maldonado Coelho Guedes, José Luiz Proença-Módena, João Pessoa Araújo Junior, Helena Lage Ferreira, Flávio Guimarães da Fonseca

**Affiliations:** 1 Departamento de Virologia, Instituto de Microbiologia Paulo de Góes, Universidade Federal do Rio de Janeiro, UFRJ, Rio de Janeiro 21941-599, RJ, Brazil; gustpdsilva@gmail.com; 2 Laboratório de Pesquisa em Virologia Animal, Departamento de Medicina Veterinária Preventiva, Escola de Veterinária, Universidade Federal de Minas Gerais (UFMG), Belo Horizonte 31270-901, MG, Brazil; alessandraalevato@hotmail.com (A.A.L.); mariaisabel.guedes@gmail.com (M.I.M.C.G.); 3 Departamento de Medicina Veterinária, FZEA-USP, Universidade de São Paulo, Pirassununga 13635900, SP, Brazil; larissambueno@gmail.com (L.M.B.); hlage@usp.br (H.L.F.); 4 Laboratório de Estudos Genômicos, Departamento de Biologia, Instituto de Biociências Letras e Ciências Exatas (IBILCE), Universidade Estadual Paulista (Unesp), São José do Rio Preto 15054-000, SP, Brazil; cintia.bittar@unesp.br; 5 Instituto de Biotecnologia (IBTEC), Universidade Estadual Paulista (Unesp), Botucatu 18607-440, SP, Brazil; gabriela.sfabiano@gmail.com (G.F.d.S.); joao.pessoa@unesp.br (J.P.A.J.); 6 Instituto de Ciências Biológicas (ICB), Universidade Federal de Minas Gerais (UFMG), Belo Horizonte 31270-901, MG, Brazil; karine_lourenco@hotmail.com; 7 Departamento de Genética, Evolução, Microbiologia e Imunologia, Instituto de Biologia, Universidade Estadual de Campinas (UNICAMP), Campinas 13083-682, SP, Brazil; jlmodena@gmail.com

**Keywords:** Brazilian Society of Virology, SBV, SBV annual meeting, human virology, veterinary virology, plant virology, invertebrate virology, basic virology, environmental virology

## Abstract

The Brazilian Society of Virology has been organizing annual meetings for 32 years now. The 32nd annual meeting, which occurred in 2021, was once again an online meeting in consequence of the issues imposed by COVID-19, even with the vaccination advances. As in the 2020 meeting, the number of attendees was high, with considerable participation by undergraduate, graduate, and postdoc students. Distinguished scientists from different countries offered high-quality conferences, and oral presentation sessions were presented by young scientists showing their newest research results. For almost five hours a day during five days, attendees discussed high-quality science related to all areas of virology. Even with the difficulties imposed by another pandemic year, the 32nd SBV annual meeting achieved its most important goal—to inspire young scientists and discuss high-quality virology research.

## 1. Introduction

The Brazilian Society of Virology (SBV) was founded in 1989, and since then, it has been a forum to conduct virology research and discuss applied virology among researchers, professors, undergraduate, and graduate students who study or work with virology in all Brazilian regions. The SBV always had a wide range of interests, with representatives from areas related to human, veterinary, plant, invertebrate, environmental, and basic virology. Annually, the SBV promotes a national congress that is held in different regions of Brazil each time.

The 31st SBV National Congress, in 2020, was in a completely online format, for the first time since the society was created, due to the COVID-19 pandemic. Even in such an unusual format, the event was a great success, gathering 921 attendees [1]. The high interest was driven by the pandemic. The Brazilian virology community worked hard and fast, moving efforts to help in the different fronts, responding to the pandemic-imposed threats with 369 poster presentations.

Since 2021, SBV has been promoting monthly virtual meetings for the discussion and presentation of talks focused on virology topics of interest for most of its members. These meetings are free, and non-SBV members are encouraged to participate. A special meeting was dedicated to talks from SBV members who showed distinguished participation in answering COVID-19 pandemic issues.

The 32nd SBV National meeting was once again conducted virtually in 2021. A total of 761 attendees, including professionals, undergraduate, and graduate students, participated in the event, and students represented 86.3% of the attendees. During five consecutive days, high-quality virology research was shared, with seminal presentations of researchers from different countries and from distinct Brazilian regions sharing new data and amazing lectures. As expected, SARS-CoV-2 was the prevalent theme responding to the largest number of conferences (66% of the total conferences) and talks (40% of the total talks, including roundtables and oral presentations). The high quality of presentations and the great number of students gathering showed that the goals of SBV were achieved in this 32nd annual meeting.

## 2. Scientific Program of the 32nd SBV Annual Meeting 

The scientific program of the 2021 SBV annual meeting included seven plenary conferences, of which four were “state-of-the-art” talks, three were technical conferences, two were roundtables, and six were oral presentation sections, with a total of 51 speakers. Moreover, during the event, six sections of e-poster presentations, covering human, plant, veterinary, invertebrate, environmental, and basic virology, were presented. A total of 315 e-posters were discussed on a real-time platform.

For conferences, roundtables, and the Hélio Gelli Pereira (HGP) Award presentations, the event had the collaboration of 20 chairs, and e-poster evaluation was performed by 40 researchers from all areas of virology. The aid of these highly engaged virologists was crucial for the success of the event.

Most of the conferences revealed up-to-date data results, including unpublished data, from research recently developed concerning human, veterinary, plant, invertebrate, environmental, and basic virology. As expected, SARS-CoV-2 and COVID-19 studies were the predominant themes, concerning all the viral and pandemic aspects.

Of the 51 speakers, 39 were from Brazil, 1 from Germany, 1 from China, 1 from Scotland, 1 from the United Kingdom, 1 from Mozambique, and 7 from the USA.

Details about the 32nd meeting could be found at https://www.even3.com.br/cbv2021 (accessed on 18 February 2022) [2] and on the SBV website https://sbv.org.br/sbv (accessed on 18 February 2022) [3].

### 2.1. Attendants of the Meeting

**Figure 1:**
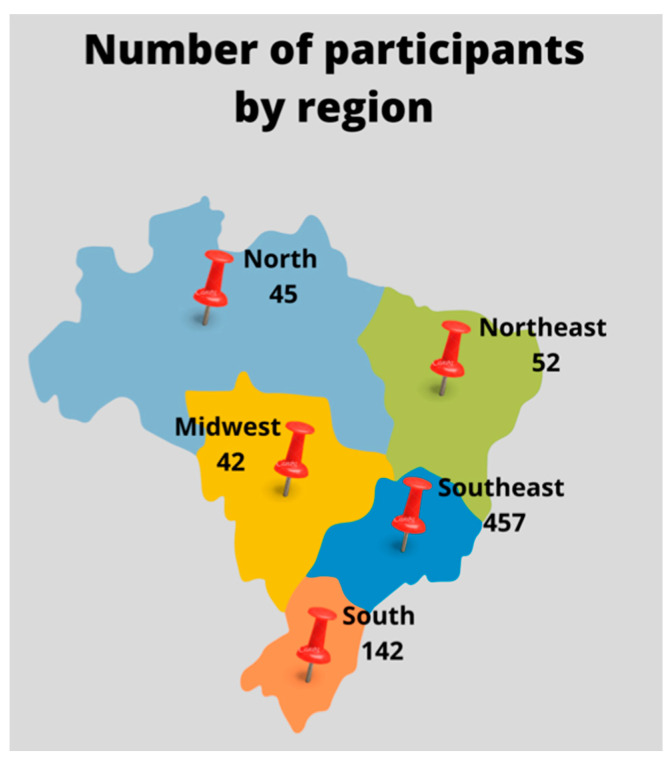
Brazilian map showing the distribution of the 738 Brazilians attendees at the 32nd SBV Annual Meeting. Each Brazilian region is represented by a different color and the number of attendees per region is shown. In total, 23 attendees were foreign and are, therefore, not represented on the map.

**Figure 2:**
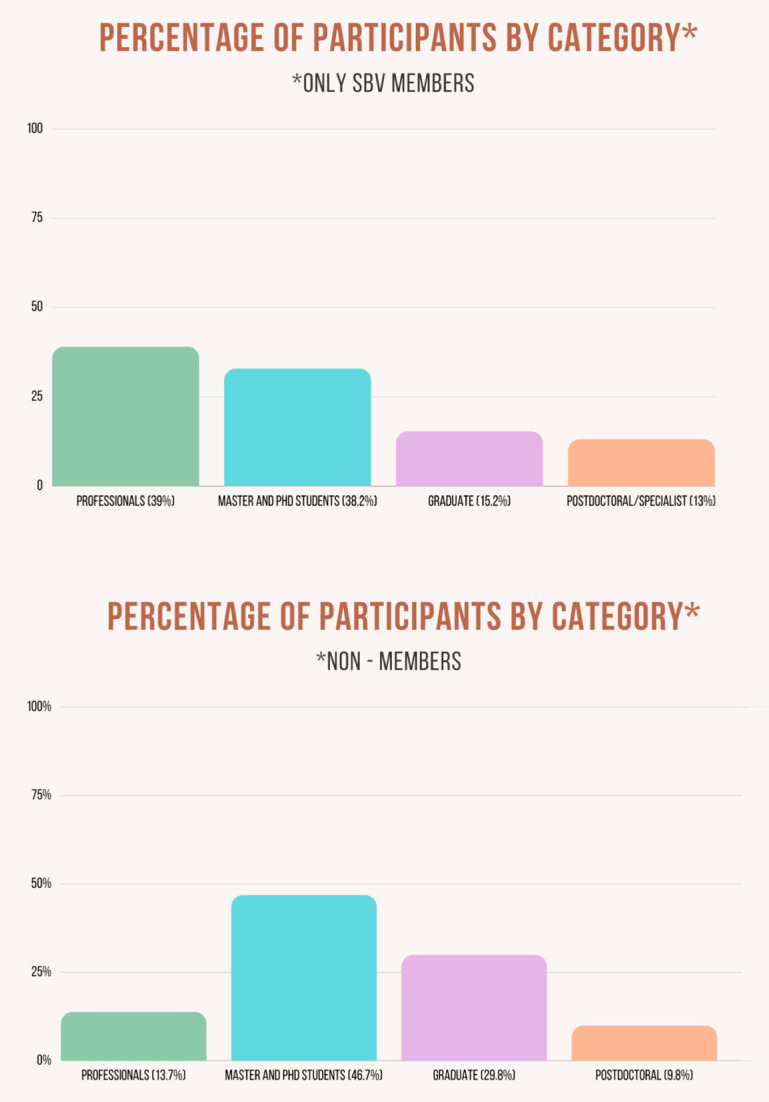
Percentage of attendees by categories. Panel superior shows attendees members of the SBV society and panel inferior the non-members attendees. * for SBV members in left panel and non-members in right panel.

The 32nd SBV annual meeting had 761 participants, including professionals, undergraduate, and graduate students from all Brazilian regions (Figure 1) and other countries. From this total, 173 were SBV members. Professionals represented 39%, and students from all categories represented 61% of the SBV members participating in the meeting (Figure 2). Among non-members of SBV, 13.7% were professionals, 9.8% were post-docs, 46.7% were Master’s and PhD students, and 29.8% were undergraduate students (Figure 2). The high adherence of students from all levels (86.3%) is a substantial achievement, showing the impact of this annual meeting on young virologists from distinct parts of Brazil. Women represented 69% (521/761) of all attendees of the 32nd SBV meeting, showing the rising contribution of women in life sciences in Brazil nowadays. However, men represented the majority of speakers in the plenary talks, which reiterates the need for positive actions to permit women scientists could have their merit recognized and represented in future events.

### 2.2. Scientific Program

The 32nd SBV meeting event was held from 19 to 23 October 2021, with activities starting in the afternoon and finishing at early night (Table 1). During an average of 4 h and 53 min per day, conferences, roundtables, oral presentations, e-posters, and technical conferences were presented in real time for attendees. The questions from the audience were posted into chats for the speakers. Discussions and answers with attendees were stimulated using chats.

### 2.3. Conference Speakers and Presentations

The opening conference was presented by Dr. George F. Gao. Dr. Gao is the head of the Chinese Center for Disease Control and Prevention and Director and Professor of CAS Key Laboratory of Pathogenic Microbiology and Immunology from the Institute of Microbiology of Chinese Academy Science, Beijing, China. His research interests include enveloped virus entry and release, as well as host jump, focusing on influenza virus interspecies transmission, structure-based drug design, and structural immunology. With a large experience in virus ecology, especially in influenza virus from wild birds or live poultry markets, and bat-derived virus, Dr. Gao presented an excellent talk, entitled “From SARS to COVID-19: challenges of coronaviruses”, covering a wide range of molecular aspects of virus–host ecological aspects and showing a wide panorama of SARS-CoV-2 emergence.

The first state-of-the-art conference, held on the second day, was held by Dr. Stephen Abedon, who is a Senior Researcher and Professor of Microbiology from the Ohio State University, Columbus, USA, and his main research interests focus on evolutionary ecology, pharmacology, biofilm interactions, and history of bacteriophages. Professor Abedon presented the lecture “Contemplating Phage Therapy as a Phage Ecologist”, showing an updated and global vision of phage therapy and highlighting the ecologic role of phages.

The conference was followed by a roundtable on SARS-CoV-2 with Dr. Michel Nussenzweig and Dr. Peter Palese. Dr. Nussenzweig, who is a Full Professor at The Rockefeller University, New York, USA, works with the human immune response since the beginning of his career, with great contributions to the area. He is renowned for the discovery that dendritic cells are antigen-presenting cells. Dr. Nussenzweig discussed “Human immune responses to SARS-CoV-2 infection and vaccination”, providing the audience with a noteworthy and complete panel of the theme. Dr. Palese, Professor of Microbiology and the Chair of the Department of Microbiology at the Icahn School of Medicine at Mount Sinai, New York, USA, held a remarkable lecture entitled “COVID-19 vaccine based on a Newcastle disease virus (NDV) vector”. Professor Palese has important contributions in RNA-containing viruses, with a special emphasis on influenza viruses, and has established the first genetic maps for influenza viruses and defined the mechanism of neuraminidase inhibitors (which are now FDA-approved antivirals). In recent years, most of the efforts by Dr. Palese and his collaborators at Mount Sinai have been involved directly in developing a universal influenza virus vaccine. However, since the beginning of the pandemic, there has been a shift in the main area, as work on COVID-19 has become central to the efforts of his group.

On 23 October, there were two “state-of-the-art” talks. The first was given by Dr. Pablo Murcia, who is a Full Professor of the MRC-University of Glasgow Center for Virus Research, College of Medical, Veterinary and Life Sciences, from the University of Glasgow, Glasgow, Scotland, where he teaches integrative virology, and his main research focus is the molecular and evolutionary mechanisms of host switching and viral emergence. Professor Murcia presented the lecture “Rhinovirus interference in SARS-CoV-2 infection”, making important contributions to understand how the previous infection with rhinovirus can impact COVID-19 patients.

Following this, Dr. George Lomonossoff presented an interesting lecture entitled “Pushing the envelope: expanding the range of virus-like particles produced in plants”, providing the audience with the progress of his group in the production of SARS-CoV-2 virus-like particles (VPLs) and how the structural proteins of the virus may dimerize. Dr. Lomonossoff is an Honorary Professor at the Universities of East Anglia and Nottingham and coordinates an important research group at John Innes Center, Norwich, United Kingdom, whose research focus has been the molecular biology of RNA plant viruses and their use in bio- and nanotechnology. He has particularly focused on the production of VLPs in plants being responsible for the development of plant-made pharmaceuticals/vaccines that have been used worldwide and are currently being utilized by the Norwich and Medicago companies.

Dr. Jesse Bloom, an Associate Professor of the Fred Hutchinson Cancer Research Center, Division of Basic Sciences and Computational Biology Program and from the Department of Genome Sciences and Department of Microbiology from the University of Washington, Washington, USA, gave the fourth state-of-the-art lecture of the meeting. Professor Bloom presented the noteworthy talk “Interpreting the evolution of SARS-CoV-2”, presenting for the audience a clear and clever vision of how human coronaviruses evolve.

Dr. Jan Felix Drexler, from the Charité Universitätsmedizin, Berlin, Germany, presented the last conference of the meeting, entitled “Emerging viruses from animal reservoirs”. The focus of Dr. Drexler’s research is the study of epidemiology and evolutionary biology of emerging viruses, with major achievements in the development of affordable tests for viruses such as HIV, hepatitis C virus, yellow fever virus, as well as in the uncovering of the zoonotic origins of major human viruses, such as mumps, hepatitis A and hepatitis B virus, and the elucidation of key aspects of the epidemiology of the Latin American Zika virus outbreak. He gave a great speech, enthusing the audience with his deep knowledge about the theme.

Three technical conferences were presented during the event. The first was about SARs-Cov-2 sequencing using distinct NGS platforms. It was presented by Dr. Vanessa Galdeno from Síntese Biotecnologia, SP, Brazil, with the title “Updating on SARS-CoV-2 VOCs detection by Illumina and Oxford Nanopore platforms”. The second technical conference was presented by Dr. Thiago Medeiros (Genetic Testing Solutions Division, Thermo Fisher, São Paulo, SP, Brazil) who addressed Thermo Fisher’s participation in the COVID-19 pandemic, with the title “Serving science in the battle against COVID-19 and beyond”. Finally, QIAGEN Brazil presented the lecture “Digital PCR–The third generation of PCR at the service of applications in virology”, presented by MSc. Arthur Silva.

The conference chairs were Dr. Felipe Naveca (Fiocruz, Manaus, AM, Brazil), Dr. Sergio de Paula (Universidade Federal de Viçosa-UFV, Viçosa, MG, Brazil), Dr. Juliane Deise Fleck (Universidade Feevale, Nova Hamburgo, RS, Brazil), Dr. Iranaia Assunção Miranda (Universidade Federal do Rio de Janeiro, Rio de Janeiro, RJ, Brazil), Dr. Gustavo Acrani (Universidade Federal da Fronteira Sul-UFFS, Passo Fundo, RS, Brazil), Dr. Paula Rahal (IBILCE-Universidade Estadual Paulista-UNESP, São José do Rio Preto, SP, Brazil), Dr. Eurico Arruda (Faculdade de Medicina de Ribeirão Preto, USP, São Paulo, SP, Brazil), Dr. Tatiana Domitrovic (UFRJ, Rio de Janeiro, RJ, Brazil), and Dr. Daniel Ardisson-Araujo (Universidade Federal de Santa Maria–UFSM, Santa Maria, RS, Brazil and Universidade de Brasilia–UnB-DF, Brasília, DF, Brazil).

### 2.4. Roundtables

A roundtable dedicated to highlighting the exceptional work carried out by young virologists, called “Young Inspiring Researchers”, was presented on the first day of the meeting. The aim of this roundtable was to stimulate young scientists showing them the high-quality research conducted by young Brazilian researchers. In the end, it was possible to conclude that, in spite of the major challenges imposed for science in Brazil, a new generation of researchers is emerging with new ideas and motivation, two essential ingredients for scientific discovery. Dr. José Luiz Proença Módena (Biology Institute, Universidade Estadual de Campinas, Unicamp, Campinas, SP, Brazil) and Dr. Maria Isabel Maldonado Coelho Guedes (Universidade Federal de Minas Gerais, UFMG, Belo Horizonte, MG, Brazil) were the chairs. Dr. Rafael Elias Marques, from the Centro Nacional de Pesquisa em Energia e Materiais (CNPEM), Campinas, SP, Brazil, presented the lecture “The structure of Mayaro virus”. Dr. Alice Freitas Versiani, from University of Texas Medical Branch, Galveston, TX, USA and Faculdade de Medicina de São José do Rio Preto (FAMERP), São José do Rio Preto, SP, Brazil, discussed “Virus nanotechnology: new frontiers for diagnostic and vaccine tools”. Dr. William Marciel de Souza, from the Department of Microbiology and Immunology at the University of Texas Medical Branch, Galveston, TX, USA, addressed “Epidemiology, genomics, and pathogenesis of arboviruses and SARS-CoV-2”. The cutting-edge research areas and the high quality of the work presented by the three “Young Inspiring Researchers” showed that the new generation of Brazilian researchers are well prepared to face the challenges that scientific work in Brazil, and elsewhere, present.

### 2.5. Abstracts, Oral Presentations, and Helio Gelli Pereira Award

**Figure 3:**
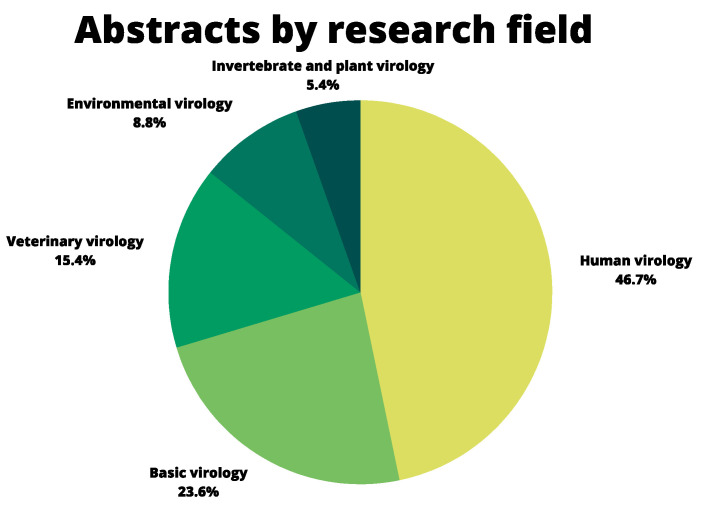
Area of interest of the abstracts presented in the 32nd SBV Annual Meeting.

A total of 353 abstracts were approved to be presented in the meeting. Human virology showed the greatest abstract participation, representing 46.7% of the total number of posters and followed by basic virology (23.6%), veterinary (15.4%), environmental (8.8%), and plant and invertebrate virology (5.4%) (Figure 3).

Among the 353 abstracts, 31 studies were selected to be presented as short oral presentations. The meeting hosted six oral presentations sections, two for human virology, with ten students in total, one of veterinary virology (with five students), one of basic virology (with five students), one of plant and invertebrate virology (with six students), and one of environmental virology (with five students) (Table 2). The studies selected for oral presentations were performed in 16 independent institutions, 14 from 3 distinct Brazilian regions, one from Mozambique, Africa (FCA), and one from the USA (Cornell University).

A scientific committee composed of 14 virology researchers from the five independent areas (human, veterinary, plant and invertebrate, environmental, and basic virology) selected the best presentation in each section of oral presentation. The winners of the best presentation of each section are highlighted in Table 2 with “*”.

All of the approved abstracts were presented as e-posters. The e-posters stayed available online during all meetings. E-posters were evaluated by a special scientific committee composed of 40 virologists. The 32nd SBV organizer committee would like to thank the great efforts and contributions of the poster analysis by the scientific committee.

During each SBV meeting, graduate and undergraduate students can apply for the Helio Gelli Pereira (HGP) Award. HGP confers one award for each category (undergraduate or graduate) with the best complete scientific article chosen by a scientific committee composed of six distinguished virologists. In this year’s competition, articles were submitted to a specific committee composed of the professors Dr. Luciana Barros de Arruda (UFRJ, Rio de Janeiro, RJ, Brazil), Dr. Iranaia Assunção Miranda (UFRJ, Rio de Janeiro, RJ, Brazil), Dr. Sergio de Paula (UFV, Viçosa, MG, Brazil), Dr. Tatiana Domitrovic (UFRJ, Rio de Janeiro, RJ, Brazil), Dr. Eurico Arruda (USP, São Paulo, SP, Brazil), and Dr. Abelardo Silva (UFV, Viçosa, MG, Brazil). Six articles were selected to be presented orally during the SBV meeting when the award committee chose the best articles/presentations in each category (undergraduate and graduate student) (Table 3).

HGP Award for the undergraduate-student category was given to Yasmin F. V. P. de Souza (UNICID, São Paulo, SP, Brazil) for the study entitled “Enteric adenovirus epidemiology from historical fecal samples in Brazil (1998–2005): Pre-rotavirus vaccine era”. The graduate-student award was conferred to Msc. Lilian Gomes de Oliveira (USP, São Paulo, SP, Brazil), who presented “Gas6 drives Zika virus-induced neurological complications in humans and congenital syndrome in immunocompetent mice”. HGP Award is supported by the American Society for Microbiology (ASM), the scientific journal *Viruses*, published by MDPI, and SBV. In 2021, ASM granted one eBook and one-year membership for the winners. *Viruses* offered a full waiver for publication by both research groups awarded, in the case of acceptance by *Viruses* referees and editors. In Brazil, most of the research institutions and universities do not support open access publication fees. Thus, these partnerships with *Viruses* represent a great incentive for students and their research groups.

## 3. Conclusions

For the second time, the 2021 SBV annual meeting was completely in an online format, due to the COVID-19 pandemic. Even with the difficulties imposed by a completely online event, a considerable level of participation was achieved, and high-quality virology science from all areas of virology was discussed by attendees. SARS-CoV-2 was, as expected, the predominant theme, showing the important effort made by researchers to answer the demand of the COVID-19 pandemic.

The high number of attendees, especially graduate and undergraduate students from all the Brazilian regions and from other countries, may reflect the advantages imposed by an online event, which provides a lower attendance fee for the attendees. With the enormous difficulties that scientific researchers currently face in Brazil, especially in financial support, this model of meeting allows more researchers to participate.

## Figures and Tables

**Table 1:** Scientific programming schedule of the 32nd Brazilian Society of Virology Congress.

Activities	Subject
19 October	
Opening session	Welcome to the participants
Conference 1	From SARS to COVID-19: challenges of coronaviruses
Roundtable 1	Young Inspiring Research
Beer and memories	Former presidents and their stories
20 October	
Poster presentation 1	Human virology
Poster presentation 2	Basic virology
Oral presentation 1	Invertebrates and plants virology
Oral presentation 2	Veterinary virology
Technical conference 1	Updating SARS-CoV-2 VOCs detection by Illumina and Oxford Nanopore platforms
State-of-the-art 1	Contemplating phage therapy as a phage ecologist
Roundtable 2	SARS-CoV-2
21 October	
Poster presentation 3	Human virology
Poster presentation 4	Invertebrates and plants virology
State-of-the-art 3	Rhinovirus interference in SARS-CoV-2 infection
Technical conference 2	Serving science in the battle against COVID-19 and beyond
Oral presentation 3	Human virology oral presentation
Oral presentation 4	Environmental virology oral presentation
22 October	
Oral presentation 5	Basic virology
Oral presentation 6	Human virology
Technical conference 3	Digital PCR–The third generation of PCR at the service of applications in virology
State-of-the-art 4	Interpreting the evolution of SARS-CoV-2
Award session	Hélio Gelli Pereira Award presentations
23 October	
Closing conference	Emerging viruses from animal reservoirs
Awards announcement	Announcement of the best poster and oral presentations and Hélio Gelli Pereira Award
SBV general assembly	Closing session of the 32nd SBV meeting

**Table 2:** Oral presentations at the 32nd Brazilian Society of Virology Congress.

First Author and Institution	Title
**Invertebrates and Plants Virology**	
Matheus Iuri Frühauf (UFPEL/RS)	Protocols for the development of cell cultures from eggs and larvae of *Apis mellifera* species [4]
Ethiane Rozo dos Santos (UFSM/RS)	A unique iflavirus was found infecting four pentatomid species and also their associated parasitoid wasp [5]
Fabricio da Silva Morgado (UnB/DF) *	**Differential lipid droplet dynamics in insect cells during abortive and successful baculovirus infections [6]**
Alex Moura da Silva (UFRJ/RJ)	Characterization of a set of genes identified in the cotton transcriptome and its expression profile in the early stages of cotton blue disease [7]
Fernanda Barreiro Brito (UFRJ/RJ)	Overexpression of arginyl t-RNA transferase induces resistance against plant RNA viruses [8]
Baltazar do Azarento Izabel Chipiringo (FCA-UNIZAMBEZE/Moçambique)	Emergence of a bipartite begomovirus in Mozambique and its recombination-driven adaptation to legume crops [9]
**Veterinary Virology**	
Maureen Hoch Vieira Fernandes (Cornell University)	A virulent and pathogenic infectious clone of *Senecavirus A* [10]
Carolina Alvarenga Turini (UFMG/MG)	Expression, biochemical characterization and purification of equine chorionic gonadotropin (ECG) produced by recombinant *Vaccinia virus* wr in mammal cells [11]
Larissa Mayumi Bueno (USP/SP)	Detection of Alphacoronaviruses in seven bat species (*Chiroptera*) in Brazilian domains [12]
Mathias Martins (Cornell University) *	**Age-related susceptibility of ferrets to SARS-Cov-2 infection** [13]
Vanessa Haach (UFRGS/RS)	Immunogenicity of a trivalent virosome-based influenza vaccine in pigs [14]
**Human Virology 1**	
Noilson Oliveira (USP/SP)	Metagenomic analysis of the purified virome in hypertrophic human tonsils [15]
Thais Melquiades de Lima (USP/SP)	SARS-CoV-2 in tonsils and adenoids from asymptomatic children [16]
Laise Rodrigues Reis (IRR/Fiocruz/MG) *	**Characterization of immune response in the duration of 17DD-YF vaccine immunity in children and adults of areas without circulation of *Yellow fever virus*** [17]
Cíntia Bittar (Unesp-Ibilce/SP)	Screening of SARS-CoV-2 variants through a Sanger sequencing strategy [18]
Leonardo Cardia Caserta (Cornell University/USA)	Vaccine breakthrough infections and transmission of SARS-CoV-2 B.1.617.2 (Delta) variant among fully vaccinated individuals [19]
**Environmental Virology**	
Natalia Ingrid Oliveira Silva (UFMG/MG)	Investigation of SARS-CoV-2 circulation in urban bats of Minas Gerais state (2020–2021) [20]
Paloma Cavalcante Cunha (UFV/MG) *	**Proposal of a phage cocktail for sulfate-reducing bacteria biofilms control in environments related to the oil and gas industry** [21]
Tatiana Prado (Fiocruz/RJ)	Virome in lakes of Antarctic, 2019–2020 [22]
Bruna Seixas da Rocha (Universidade Feevale/RS)	Surveillance of SARS-CoV-2 in a wastewater treatment plant in the metropolitan region of Rio Grande do Sul state [23]
Lilian Gonçalves do Nascimento (Fiocruz/RJ)	Detection and characterization of Human adenovirus in bivalve shellfish collected in Arraial do Cabo, RJ [24]
**Basic Virology**	
Isadora Alonso Corrêa (UFRJ/RJ)	Replicative comparison and neutralization capacity of SARS-CoV-2 Gamma variant and its ancestor B.1.1.28 [25]
Vinicius Bottura Apolloni (USP/SP)	AP-1 is a host factor that regulates HIV-1 envelope glycoprotein trafficking [26]
Amanda Izeli Portilho (USP/SP)	Cellular immune response to SARS-CoV-2 antigen adjuvanted by outer membrane vesicles in mice [27]
Juliano de Paula Souza (USP/SP) *	**Breastfeeding by Chikungunya virus-infected dams confers resistance to challenge in the offspring** [28]
Sharton Vinícius Antunes Coelho (UFRJ/RJ)	Contact system activation in plasma from Dengue patients might harness endothelial virus replication through the signaling of bradykinin receptors [29]
**Human Virology 2**	
Vanessa Cristine de Souza Carneiro (Fiocruz/RJ)	Herpesvirus reactivation and neurological manifestations in patients with severe COVID-19 [30]
Bruna Candia Piccoli (UFSM/RS)	Transcriptional downregulation of ACE-2 during SARS-CoV-2 infection and its correlation with SIRT-1 and ADAM-17 [31]
Fernando Luz de Castro (UFRJ/RJ) *	**Study of the immunoglobulin repertoire in SARS-CoV2 infection: isolation and short-term production of potentially neutralizing antibodies** [32]
Priscilla Paschoal Barbosa (UNICAMP/SP)	Identification of viral pathogens in patients with central nervous system infection and fatal outcome using metagenomic approach [33]
Patrícia de Melo Oliveira (UFMG/MG)	In silico analysis of SARS-CoV-2 viral signatures in the T CD8+ cell-mediated memory response [34]

* The best presentations are highlighted in the table.

**Table 3:** Oral presentation on the HGP Award.

Hélio Gelli Pereira–Award Session	
**Undergraduate category**	
Yasmin F V P de Souza (UNICID/SP) *	**Enteric adenovirus epidemiology from historical fecal samples in Brazil (1998–2005): Pre-rotavirus vaccine era** [35]
**Graduate category**	
Bruna Amaral Lapinscki (UFPR/PR)	Influenza A disease severity—a retrospective cross-sectional study, southern Brazil [36]
Juliana Aparecida Souza da Paz (UFRJ/RJ)	Viable SARS-CoV-2 found in rectal swabs of domestic animals [37]
Lilian Gomes de Oliveira (USP/SP) *	**Gas6 drives Zika virus-induced neurological complications in humans and congenital syndrome in immunocompetent mice** [38]
Mariene Ribeiro Amorim (UNICAMP/SP)	Neutralization of SARS-CoV-2 lineage P.1 by antibodies elicited through natural SARS-CoV-2 infection or vaccination with an inactivated SARS-CoV-2 vaccine: an immunological study [39]
Paula Rogovski (UFSC/SC)	*Salmonella enterica* serovar *enteritidis* control in poultry litter mediated by lytic bacteriophage isolated from swine manure [40]

* The best presentations are highlighted in the table.

## Data Availability

Not applicable.
